# Coagulation and Mental Disorders

**DOI:** 10.5041/RMMJ.10170

**Published:** 2014-10-29

**Authors:** Silvia Hoirisch-Clapauch, Antonio Egidio Nardi, Jean-Christophe Gris, Benjamin Brenner

**Affiliations:** 1 Department of Hematology, Hospital Federal dos Servidores do Estado, Ministry of Health, Rio de Janeiro, Brazil;; 2 Institute of Psychiatry, Federal University of Rio de Janeiro, National Institute for Translational Medicine (INCT-TM), Brazil;; 3 Department of Haematology, Centre Hospitalier Universitaire, Nîmes, France;; 4 Department of Hematology and Bone Marrow Transplantation, Rambam Health Care Campus and Technion Institute of Technology, Haifa, Israel

**Keywords:** Anxiety, cardiovascular diseases, depression, mental disorders, schizophrenia, thrombosis

## Abstract

The neurovascular unit is a key player in brain development, homeostasis, and pathology. Mental stress affects coagulation, while severe mental illnesses, such as recurrent depression and schizophrenia, are associated with an increased thrombotic risk and cardiovascular morbidity. Evidence indicates that the hemostatic system is involved to some extent in the pathogenesis, morbidity, and prognosis of a wide variety of psychiatric disorders. The current review focuses on emerging data linking coagulation and some psychiatric disorders.

## INTRODUCTION

The human brain critically relies on an elaborate vascular network, containing up to 100 billion vessels, a vessel for each neuron. Apart from the oxygen supply function, endothelial cells also produce instructive signals for neural development, support normal functioning, ensure maintenance, and promote reparative regeneration of the central nervous system. The brain vasculature is increasingly recognized as a key player that actively influences and directs brain development, homeostasis, and pathology.^1^ One of the major challenges of the century is to improve the understanding and treatment of psychiatric diseases. Among the physiologic systems which may be influenced by, and/or may influence, the neurovascular unit is the hemostatic system. Elucidating the cross-talk between hemostasis, thrombosis, and the fibrinolytic system and the nervous system is crucial to understand better the molecular and cellular basis of how the brain operates, but also to develop novel diagnostic and therapeutic strategies for mental illnesses. We herein review the emerging knowledge linking hemostasis and some psychiatric disorders.

## ANXIETY DISORDERS

The same stress responses that were essential in ancient times to protect the body against fatal bleeding can be elicited by emotional stimuli or professional and social stress. Fear and anxiety are important responses to stress, mediated by several neurotransmitters and hormones, including epinephrine, norepinephrine, serotonin, acetylcholine, dopamine, prolactin, and cortisol. While fear is the emotional response to real or perceived imminent threat, anxiety is anticipation of future threat.^2^

Catecholamines have a prominent role in the fight-or-flight reaction. In response to catecholamine and also to serotonin stimulus, platelets manifest a series of functional responses, such as adhesion, secretion, and aggregation.^3^ Catecholamines, particularly epinephrine, bind to the β2-adrenoreceptor, resulting in release of factor VIII, von Willebrand factor, and tissue plasminogen activator. Importantly, tissue plasminogen activator is expressed in chromaffin cells, and co-released with catecholamines by chromaffin cell stimulation.^4^ The simultaneous activation of coagulation and fibrinolysis during acute stress can be demonstrated by increased levels of D-dimer.^3^

Through α-adrenoreceptor activation, catecholamine induces vasoconstriction. During vasoconstriction plasma, but not large plasma proteins, passes easily into interstitial spaces through minute openings or pores in the capillaries. The presence of large molecules creates a large colloidal osmotic pressure gradient^5^ that increases viscosity, which contributes to a hypercoagulable state. Factor VIII activity remains elevated, even after saline reconstitution, suggesting that the intrinsic pathway is genuinely activated during acute stress.^3^

Anxiety disorders differ from transient fear or anxiety by an excessive response to stress, with a typically chronic course.^2^ In severe post-traumatic stress disorder, for example, although D-dimer levels might be normal, von Willebrand antigen and factor VIII activity are usually elevated, and platelets exhibit exaggerated reactivity to *in vitro* epinephrine/ADP stimulation mediated by the α2-adrenergic receptor.^3^,^6^ Opposed to acute stress, chronic stress seems to impair fibrinolytic activity.^3^ Abnormal fibrinolysis is reflected by increased synthesis of plasminogen activator inhibitor (PAI)-1, a major inhibitor of tissue plasminogen activator, probably as a result of serotonin and cortisol secretion.^7^,^8^ Angiotensin potentiates the production of PAI-1 stimulated by steroids,^9^ which provides a reasonable explanation for the increased thrombotic tendency and cardiovascular risk seen in patients who are both anxious and hypertensive.^3^,^10^

## DEPRESSIVE DISORDERS

Different from sadness, which is a normal response to loss, depressive disorders are disabling illnesses that may present with psychotic symptoms, such as delusions or hallucinations, and frequently co-occur with lifetime anxiety.^11^ They include major depresssive disorder and many other subtypes, such as dysthymia and premenstrual dysphoric disorder. Major depressive disorder may present with melancholic or with atypical features.^2^ Melancholia is characterized by diurnal variation of depressive symptoms, usually worse in the morning, by insomnia and anorexia, and by a hyperactive stress response. Patients with melancholia are usually anxious, less responsive to the environment, and they dread the future.

Major depression accompanied by atypical features is defined as mood improvement in response to positive events, plus at least two of the following four symptoms: significant weight gain or hyperphagia, hypersomnia, severe lethargy, also described as heavy, leaden feelings in arms or legs, and pathological rejection sensitivity. Whereas patients with melancholia usually have increased levels of corticotrophin-releasing hormone and diminished activities of the growth hormone and reproductive axes, in atypical depression hypothalamic-pituitary adrenal is typically down-regulated and corticotrophin-releasing hormone is deficient.^12^

Serotonin seems to play a major role in the pathophysiology of depression. More than 99% of the circulating serotonin is found in platelet-dense granules,^13^ and serotonin itself is a platelet agonist. In patients with recurrent depressive disorders, platelet studies have shown increased serotonin response, increased serotonin receptor density, decreased serotonin transporter binding, blunting of adenosine response, increased thrombin response, increased expression of glycoprotein 1b, P-selectin, β thromboglobulin, platelet factor 4, fibrinogen, factor V, and anionic phospholipids, as well as decreased platelet content of brain-derived neurotrophic factor and serotonin.^14^,^15^ Clot firmness and procoagulant activity of platelet-associated tissue factor may also be significantly elevated. Additionally, studies with circulating blood revealed increased fibrin formation in early diagnosed patients.^15^

Abnormalities in the fibrinolytic pathway can be also observed in depressed subjects. Because PAI-1 promoter has cortisol and insulin response, depressed individuals may have elevated levels of PAI-1 activity, even after adjusting for confounders, such as coronary heart disease, smoking, hypertension, triglyceride concentration, and body mass index.^16^ Also, serotonin has been shown to increase PAI-1 levels in endothelial cells.^17^

## SCHIZOPHRENIA

Compared to controls, first-episode psychosis patients display higher levels of markers of thrombogenesis, such as D-dimer and factor VIII, and markers of platelet activation, such as soluble P-selectin.^18^ Additionally, first-episode drug-naïve schizophrenia patients may exhibit higher cortisol and insulin levels than healthy subjects, as well as a significantly higher insulin resistance score.^19^ The PAI-1 promoter has insulin and cortisol response, and it is possible that elevated PAI-1 levels could contribute to the increased cardiovascular and thrombotic risk seen in schizophrenia patients.

A high prevalence of free-protein S deficiency has been demonstrated in a sample of schizophrenia patients. Fifteen out of 70 (22%) and none of 98 controls showed low levels of the natural anticoagulant. Except for one patient with periodontitis, no patient had clinical or laboratory evidence of inflammation. Even though it was not determined in this study if deficiency was inherited or acquired, low levels of free-protein S increased the chances of having a first-degree relative with schizophrenia by 145 times compared to controls, which suggests that, at least in some of the patients, protein S deficiency could have been inherited.^20^ Aside from having anticoagulant activity, free-protein S accelerates neutralization of PAI-1, and therefore increases fibrin clot lysis. Moreover, it also affords neuroprotection.^21^

## REPERCUSSION OF MENTAL DISORDERS ON CARDIOVASCULAR RISK

Generalized anxiety disorder, post-traumatic stress disorder, and panic disorder are strongly associated with increased risk for cardiovascular morbidity and mortality.^22^–^25^ A bidirectional relationship exists between depression and cardiovascular disease. Depression increases the risk for cardiovascular disorders such as coronary artery disease and myocardial infarction 1.5–2-fold and for stroke 1.8-fold.^26^ Likewise, in patients with coronary heart disease, depression is independently associated with cardiovascular morbidity and mortality.^27^ Besides susceptibility to blood clotting, various pathophysiological mechanisms may underlie the risk of cardiovascular disease in patients with depression, including oxidative stress, hypothyroidism, hyper-activity of the sympatho-adrenomedullary system and the hypothalamic-pituitary-adrenal axis, and reduction in arterial repair processes.^26^ Different studies suggest an increased risk for coronary artery disease not only in patients with major depression but also in those with depressive symptoms and dysphoria. In these studies, the magnitude of risk seems to follow a graded effect, with increasing risk for coronary artery disease among patients with more severe depression.^28^

Patients with bipolar disorders are known to be at high risk of premature death. A systematic review of recently published articles suggested that a multisystemic inflammatory involvement is present from the early stages, and a dysregulated inflammatory background seems to be a common factor underlying cardiovascular and bipolar disorders, with hemostasis activation paying tribute to immuno-inflammation.^29^

The increased risk for cardiovascular disease observed in schizophrenia patients has been related to inactivity, dietary habits, hyperhomocysteinemia, the high rate of substance abuse, including tobacco and illicit drug use, and alcoholism. Other risk factors may contribute to the increased risk for cardiovascular events. For example, insulin resistance, a common psychotropic side effect, may cause obesity. Additionally periodontitis is highly prevalent in schizophrenia patients due to both heavy tobacco smoking and inadequate oral hygiene. Obesity and periodontitis may cause systemic inflammation, which in turn may reduce protein S activity and increase PAI-1 levels, thus increasing thrombotic and cardiovascular risk. Increased cardiovascular risk is already observed in first-episode schizophrenia.^30^,^31^

## REPERCUSSION OF MENTAL DISORDERS ON THROMBOTIC RISK

Patients with pulmonary hypertension have more panic disorders than controls.^32^ Based on the assumption that recurrent showers of small emboli, which may give rise to pulmonary hypertension, are commonly undetected clinically, it is possible that some episodes of panic attacks might accompany undetected episodes of pulmonary thromboembolism.

Both thromboembolism and depression are common complications of neoplastic disorders. In patients with recurrent depressive disorders without neoplastic disorders, however, despite platelet activation and abnormal fibrinolysis,^33^ there is no evidence that depression per se increases the risk of thromboembolism. There are three possibilities. First, there have been no specific studies assessing the incidence of venous thrombosis in depressed patients. Second, serotoninergic antidepressants may impair platelet function and decrease PAI-1 levels, therefore reducing the thrombotic tendency.^34^ Another explanation is that even though plasma levels of interleukin-6 and tumor necrosis factor-α may be increased in patients with depression,^35^ these patients often display features of immunosuppression, such as accelerated apoptosis of both T lymphocytes and neutrophils.^36^

Schizophrenia patients may also be at increased risk of thromboembolic events. Thrombotic tendency has been usually associated with psychotropic medication and with immobility, as in restraint or catatonia. In a study conducted in restrained psychotic patients, the incidence of deep vein thrombosis was 12% in spite of prophylaxis with graduated compression stockings and subcutaneous injection of unfractionated heparin.^37^ The finding of high levels of thrombogenesis markers and platelet activation in first-episode psychosis patients suggests that mechanisms involved in the pathogenesis of psychosis might also contribute to the thrombotic tendency.^18^

## ASSOCIATION OF THROMBOTIC RISK MARKERS WITH MENTAL DISORDERS

Among all classical thrombophilias, i.e. abnormalities of blood coagulation that enhance the thrombotic risk, only an acquired one, namely antiphospholipid antibodies (APLA), has been substantially described in patients with severe mental disorders.^38^

Associations with hyperactivity and behavioral abnormalities were reported in mice exposed to human APLA, as well as in mice immunized with a major APLA cofactor, β2 glycoprotein 1. Human APLA can directly permeabilize and depolarize brain synaptosomes, with histological examination showing antibody binding to both neuronal and white matter. Immunogold electron microscopy revealed APLA reacting with myelin, mainly the major dense line formed by the cytoplasmic apposition of the oligodendrocyte plasma membrane. The effects of APLA on brain endothelial cells may influence inflammatory and hypercoagulation effects on the brain tissues. Antiphospholipid antibody-mediated endothelial cell activation is likely to disrupt the blood–brain barrier, facilitating the diffusion of pathogenic antibodies, coagulation factors, and inflammatory factors into the brain tissues. Direct effects of autoantibodies on various brain cellular components may induce the behavioral changes observed in the mice passively or actively developing the antiphospholipid syndrome.

Case reports of bipolar disorders, of schizo-affective disorder, and of schizophrenia have been convincingly argued in APLA-positive patients. In some cases, the acute onset of psychiatric symptoms even revealed a significantly sustained positivity for APLA. Some reports have also suggested that APLA may emerge as a response to antipsychotic treatment, since a high prevalence of the antibodies in antipsychotic users has been observed. A recent cross-sectional study determined the presence and a subtype of APLA in 333 psychiatric inpatients: APLA were frequently found positive, and prevalence of the antibodies was not significantly different in patients who were receiving, or not receiving, antipsychotics.^39^ Long-term prospective epidemiological cohort studies are still needed to analyze possible pathogenic implications of persistent APLA in psychiatric patients, such as worsening of the clinical symptomatology.

## TREATMENT OF MENTAL DISORDERS MAY AFFECT COAGULATION

In patients with anxiety and depressive disorders, factor VII and plasminogen activator inhibitor levels normalize after psychotherapy, indicating that improvement of psychiatric symptoms somehow reverses the procoagulant effect of these psychological states.^10^ The same may occur with pharmacological treatment. Of note, compared to nonserotoninergic antidepressants, serotoninergic antidepressants may decrease the risk of arterial occlusive events, such as myocardial infarction, but may also increase the risk for abnormal bleeding, which includes perioperative, gastrointestinal, and brain hemorrhage.^34^ The increased bleeding risk observed in some patients on serotoninergic anti-depressants has been related to platelet and fibrinolytic abnormalities. Platelets of serotoninergic antidepressant-medicated patients display a lower serotonin content and lower ADP, collagen, or epinephrine-induced aggregation.^40^ Furthermore, patients on serotonergic antidepressants appear to have fibrinogen and PAI-1 plasma levels that are similar to those of healthy controls, but lower than in depressed patients receiving non-serotonergic antidepressants.^41^

In a group of psychotic patients, plasma levels of soluble P-selectin varied significantly in the course of 1-year antipsychotic treatment, mainly between 3 and 6 months after therapy was started, but plasma levels of D-dimer and factor VIII remained elevated.^18^ Antipsychotics, especially clozapine and olanzapine, may promote weight accrual and increase the levels of insulin and triglycerides.^42^ Fat tissue stroma synthesizes PAI-1, and both insulin and triglycerides provide stimulus for PAI-1 synthesis, which may increase cardiovascular risk.

Schizophrenia patients with hyperhomocysteinemia might benefit from B-vitamin supplementation: when these patients were treated with folic acid, B_12_, and pyridoxine, clinical symptoms as measured by the Positive and Negative Syndrome Scale declined significantly.^43^ Considering that hyperhomocysteinemia is an independent risk factor for both cardiovascular disease and thromboembolism, it remains to be defined if vitamin supplementation reduces morbidity and mortality in this group of patients.

## CONCLUSION

Preliminary emerging data suggest that the hemostatic system may play either a direct or an indirect role in the pathogenesis, morbidity, and prognosis of mental disorders. Interdisciplinary efforts are mandatory to elucidate the numerous uncertainties related to the associations of thrombosis, cardiovascular disorders, and psychiatric presentations. This could potentially pave the way for refined diagnosis and improved management.

## Abbreviations:

APLA: antiphospholipid antibodies;
PAI: plasminogen activator inhibitor.
